# Indoleamine 2,3-dioxygenase expression in patients with allergic rhinitis: a case-control study

**DOI:** 10.1186/2045-7022-1-17

**Published:** 2011-12-12

**Authors:** Annika Luukkainen, Jussi Karjalainen, Teemu Honkanen, Mikko Lehtonen, Timo Paavonen, Sanna Toppila-Salmi

**Affiliations:** 1 Department of Otorhinolaryngology, University of Tampere, Tampere, Finland; 2 Allergy Centre, Tampere University Hospital, Tampere, Finland; 3 Department of Otorhinolaryngology, Kanta-Hame Central Hospital, Hameenlinna, Finland; 4 Department of Pathology, University of Tampere, Tampere, Finland; 5 Department of Otorhinolaryngology, University of Tampere, Tampere, Finland; 6 Department of Otorhinolaryngology, Kanta-Hame Central Hospital, Hameenlinna, Finland; 7 Department of Pathology, Centre for Laboratory Medicine, Tampere University Hospital, Tampere, Finland; 8 Helsinki University Hospital, Skin and Allergy Hospital, Helsinki, Finland; 9 Transplantation laboratory, Haartman Institute, University of Helsinki, Helsinki, Finland

**Keywords:** Indoleamine 2,3-dioxygenase, allergic rhinitis, birch pollen, dendritic cell, tryptophan, kynurenine, interferon gamma, leukocyte, eosinophil

## Abstract

**Background:**

Indoleamine 2,3-dioxygenase (IDO) is a tryptophan catalyzing enzyme. It has been suggested that it has a role in lower airway allergic inflammations, but its role in allergic rhinitis has not been investigated.

**Objective:**

Our aim was to evaluate the expression of IDO in the nasal mucosa of allergic rhinitis patients allergic to birch pollen during peak exposure to birch pollen allergen and compare it to non-atopic patients.

**Methods:**

IDO expression was immunohistochemically evaluated from nasal specimens obtained in- and off-season from otherwise healthy non-smoking volunteers both allergic to birch pollen (having mild or moderate allergic rhinoconjunctivitis) and non-allergic controls. **Results: **The IDO expression levels were low in healthy controls and remained low also in patients allergic to birch pollen. There were no differences in the expression of IDO in- and off-season in either healthy or allergic subjects.

**Conclusions:**

There is a controversy in the role of IDO in upper and lower airways during allergic airway disease. It seems that IDO is associated to allergic inflammations of the lower airways, but does not have a local role in the nasal cavity at least in mild or moderate forms of allergic rhinitis.

## Introduction

Indoleamine 2,3 dioxygenase (IDO) is an intracellular enzyme that initiates the first and rate-limiting step of tryptophan breakdown along the kynurenine pathway [1]. IDO is widely expressed in a variety of cell types including leukocytes and tumour cells [2]. Initially the role of IDO was thought to be mainly antimicrobial by reducing the availability of the essential amino acid tryptophan in the inflammatory environment [3]. In the past years, IDO has emerged as an important regulator of the immune system; however, it is not known whether local IDO activity is beneficial or detrimental to inflamed tissues. IDO is induced by interferon γ (IFN-γ) and other inflammatory cytokines during inflammation or as a consequence of normal tissue function [4]. IDO suppresses T cell activity and promotes T cell tolerance to further antigenic challenges, by promoting the differentiation of naïve CD4 T cells into regulatory T cells, putatively by regulation by dendritic cells [5-10]. IDO seems to serve as a negative feedback loop or is not essential for Th1 responses, but plays a distinct role in up-regulating Th2 dominant immune responses [2,11]. Moreover, IDO has also been shown to down-regulate Th2 responses [12]. The role of IDO in the modulation of allergic airway inflammation has recently been investigated [13-16].

Our objective was to observe IDO expression levels in the nasal mucosa of allergic rhinitis patients allergic to birch pollen in relation to exposure to birch pollen allergen and compare it to healthy controls.

## Materials and methods

### Subjects

This study is a case-control study. It was carried out at the Department of Otorhinolaryngology, Tampere University Hospital, Finland and has been approved by the Hospital's Ethical committee. Written informed consent was obtained from all patients. The subjects were Caucasian. Patients were either atopic with allergic rhinoconjunctivitis symptoms, or non-atopic. Moreover, the patients did not have other diseases such as asthma. The diagnosis of birch pollen-induced allergic rhinitis was based on a history of seasonal allergic rhinitis during spring, clinical examination, and skin prick test positivity. Characteristics of the subject groups are shown in table 1. Specimens were taken from the nasal cavity. Biopsies from the anterior edge of the inferior turbinate were obtained with Fokkens' forceps under local anaesthesia. Specimens were taken during both winter (off season) and during peak allergen exposure in spring (in season, natural allergen exposure). Patients were not allowed to use their medication (antihistamine and/or nasal corticosteroids) for a minimum of 5 days before nasal biopsies were taken.

**Table 1:** Patient characteristics

	Control n = 15	Atopy n = 12	P value
Age			
median	23	24.5	NS
min-max	21-36	21-34	
No. of male sex	4	5	NS
Peripheral blood eosinophils			
median	0.23	0.15	NS
Q1-Q3	0.085-0.295	0.12-0.20	
% of peripheral blood eosinophils			
median	3.0	2.5	NS
Q1-Q3	2.0-4.0	2.0-4.0	
S-IgE			
median	27.0	182.0	< .001
Q1-Q3	18.5-58.5	67.0-410.0	
IgE birch			
median	< .35	62.5	< .001
Q1-Q3	< .35- < .35	12.0-161.0	
IgE timothy grass			
median	< .35	4.75	< .001
Q1-Q3	< .35- < .35	1.6-.6.1	
No. subjects having SPT positivity to any basic allergen	0	12	< .001
birch	0	12	< .001
timothy grass	0	9	< .001
other pollen	0	12	< .001
animal dander	0	9	< .001
house dust mite	0	0	NS
other basic allergens	0	0	NS
Biopsies were taken during			
winter	11	8	NS
spring	7	11	.019
both	3	7	NS

### Sample staining

Nasal specimens were immediately snap-frozen in liquid nitrogen and stored at -80°C until analysis. Tissue samples were stained with hemalaun-eosin to calculate the number of mucosal leukocytes and eosinophils/mm^2 ^and to evaluate the inflammation score (0 = no inflammation, 1 = mild, 2 = moderate, 3 = severe inflammation) For light microscope evaluation, immunoperoxidase staining was used and specimens were cut into 3-5 μm thick frozen sections on Superfrost Plus microscope slides. (Menzel-Gläser, Braunschweig, Germany). Frozen sections were fixed with formalin during 45 minutes. Fully automated immunostaining was performed by Ventana BenchMark LT Automated IHC Stainer (Ventana Medical System, Arizona, USA). Ultraview Universal DAB detection kit (catalogue No. 760-500, Ventana Medical System, Arizona, USA) was used. For epitope retrieval CC1: Tris -EDTA buffer pH 8.0 (catalogue No 950-124, Ventana) was used at 95°C to 100°C for 8 minutes. Endogenous peroxidase was blocked with UV-Inhibitor 3% H202 (Ventana) for 4 minutes at 37°C. Tissue slides were rinsed between steps with Ventana Tris-based Reaction buffer (catalogue No. 950-300, Ventana). Slides were incubated at 37°C for 32 minutes with mAb anti-Indoleamine 2,3 dioxygenase (1:200, clone MAB5412, Chemicon International Inc., USA) followed by application of Ventana Ultraview HRP Universal Multimer (8 minutes at 37°C). Diaminobenzidine (DAB) was used as a chromogen and haematoxylin as a nuclear stain. Known positive tissue samples (from coeliac or inflammatory bowel disease) were also used to confirm the staining reliability of all separate staining patches [17,18]. The specificity of immunohistochemistry was controlled by omitting the primary antibodies or replacing them with irrelevant antisera.

### Light microscopic evaluation

Sections were examined with a Leica DM 2000 light microscope (Leica Microsystems GmbH, Wetzlar, Germany) by two independent observers blinded to the experimental conditions. In a sample, there was either moderate or strong immunoreactivity of all epithelial cells or no epithelial positivity at all. Thus, the results were expressed as IDO positive epithelium or IDO negative epithelium. The percentage of IDO positive mucosal leukocytes was counted.

### Data analysis

Statistical analysis was carried out by SPSS Base 15.0 Statistical Software Package (SPSS Inc., Chicago, IL, USA). Data is expressed as medians or means. For comparisons, the results were analysed by, Fisher's exact (discrete) or Kruskal Wallis and Mann Whitney U tests (continuous). For pair-wise comparisons McNemar's and Wilcoxon tests were used. Two-tailed P-values of < 0.05 were considered statistically significant.

## Results

**Figure 1:**
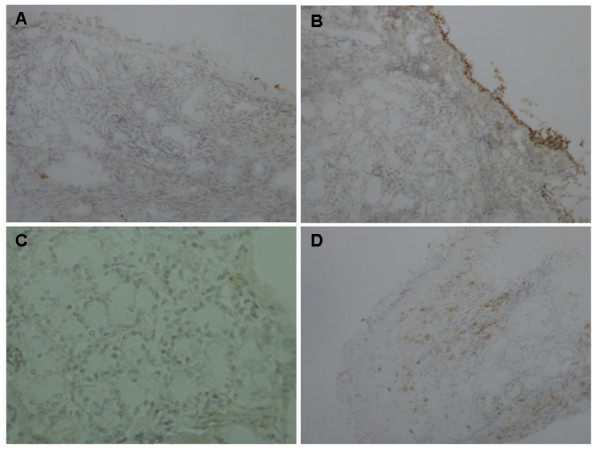
**The expression of indoleamine 2,3-dioxygenase (IDO) in biopsies from the nasal cavity**. (A) Nasal mucosa taken from a patient with allergic rhinitis off-season (during winter). No epithelial or leukocyte expression of IDO. (B) Specimen from another patient with allergic rhinitis, taken off-season, has epithelial IDO^+ ^and leukocyte IDO^-^. (C) The same allergic rhinitis patient as in frame A, did not display any IDO expression in the specimen taken in-season (during spring). (D) Specimen from a healthy patient, taken off-season, shows epithelial IDO^- ^and leukocyte IDO^+^. Original magnification 200× (A, B, D) and 400× (C).

IDO was expressed in the vicinity of the Golgi apparatus of epithelial cells, but not on the supraepithelial mucus. IDO was additionally expressed weakly in submucosal leukocytes and in intraepithelial glands. When observing the nasal biopsies, the number of specimens having positive epithelial IDO staining was not associated with atopy during either winter or spring (P > 0.05 by Fisher's test, Table 1, Figure 1). The percentage of IDO positive leukocytes did not associate with atopy in specimens taken during either winter or spring (P > 0.05, by Kruskal-Wallis test, Table 2, Figure 1). Nor did it associate with the expression of IDO in the epithelium (P > 0.05, by Kruskal-Wallis test, data not shown). The subjects did not have changes in the epithelial IDO expression or in the percentage of IDO positive leukocytes when comparing specimens taken during winter and spring from the same individuals (P > 0.05, by Wilcoxon test, Table 2). The median number of mucosal eosinophils was significantly higher in atopic than in non-atopic subjects only during symptomatic spring (P = 0.044, by Kruskal-Wallis and Mann Whitney U test, table 2), whereas the percentage of eosinophils did not significantly differ between atopic and non-atopic subjects (P > 0.05, by Kruskal-Wallis test, table 2). Interestingly, during spring the number of mucosal leukocytes and the percentage of IDO positive leukocytes correlated significantly (P < 0.05, r = 0.46, by Spearman rank correlation test, data not shown).

**Table 2:** Characteristics of nasal specimens

	Control winter n = 7	spring n = 11	P value^1^	Atopy winter n = 11	spring n = 8	P value^1^	P value^2^
Symptom score							
median	0	0	NS	0	2.0	.031	w NS*
Q1-Q3	0-0	0-0		0-0	1-2		s .003*
Inflammation score							
median	1.5	1.5	NS	1.0	1.5	NS	w NS*
Q1-Q3	1-2	1-2		1-2	1-3		s NS*
No. of leukocytes/mm^2^							
median	640	1328	NS	1344	1328	NS	w NS
Q1-Q3	384-896	1280-1376		1120-1408	992-1760		s NS
No. of eosinophils/mm^2^							
median	96	168	NS	144	248	NS	w NS
Q1-Q3	48-144	112-224		128-160	192-448		s .044
Percentage of eosinophils							
median	14.3	12.5	NS	12.0	25.5	NS	w NS
Q1-Q3	12.5-16.1	8.8-16.3		9.1-12.5	12.9-29.0		s NS
Percentage of IDO^+ ^eosinophils							
median	0.0	0.0	NS	0.0	0.0	NS	w NS
Q1-Q3	0.0-0.0	0.0-0.0		0.0-2.5	0.0-5.5		s NS
No. specimens with							
epithelial IDO^+^	2	2	NS	1	2	NS	w NS* s NS*

Patient characteristics and results of specimens taken from the nasal cavity. Control = mucosa from inferior turbinate from patient without inflammation of nasal mucosa and without sinonasal disease, P value^1 ^= by pair-wise comparison between winter and spring by Wilcoxon test; P value^2 ^= comparison between control and atopy groups either in winter (w) or in spring (s) by *Fisher's exact test (discrete) or Kruskal Wallis test (continuous).

As expected, atopics had significantly increased serum IgE levels (p < 0,001, by Kruskal-Wallis, Mann Whitney U). Atopic subjects had a significantly increased median symptom score during symptomatic spring in comparison to asymptomatic winter (P = 0.031, by Wilcoxon test, table 2), whereas in the non-atopic subjects, the median symptom score in winter and spring did not vary (P > 0.05, by Wilcoxon test, table 2). Accordingly, the median symptom score was significantly higher in the atopic than in the non-atopic group during spring but did not differ during winter (P = 0.003, P > 0.05, respectively, by Fisher's exact test, table 2).

## Discussion

We aimed to evaluate IDO expression in two well defined human phenotypes: controls and their atopic counter-parts. Skin prick tests (SPT) were performed on all study subjects to confirm diagnosis. The control and atopic groups did not differ in terms of patient number, age, sex, peripheral blood eosinophils, percentage of peripheral blood eosinophils. (p > 0,05 Kruskal-Wallis, Mann Whitney U) In comparison to controls, atopics had significantly higher serum IgE levels as well as significantly higher IgE levels against allergens to which the subjects were allergic. Atopics and controls differed in their symptom score only during the spring (in season, natural allergen exposure). Surprisingly, there were no differences between inflammation score, leukocyte numbers, percentages of eosinophils, IDO+ in epithelial cells and leucocytes during winter (off season) and spring. Atopics had a higher number of eosinophils during spring only.

We observed that in the nasal cavity, expression of IDO was low and did not differ between atopic and non-atopic groups. Moreover IDO expression did not fluctuate in the same individuals between symptomatic spring and asymptomatic winter season. We have also previously shown that the role of the nasal epithelium in birch pollen atopy is important [19]. In contrast others have shown that IDO might have a role in atopy. Paveglio et al. very recently showed in a transgenic mouse model that over-expression of IDO in the lungs might cause an anti-asthmatic effect by diminishing proliferation, numbers and cytokine production of CD4 + T cells [20]. Others have also previously shown that after *in vivo *aeroallergen exposure, serum IDO activity was increased in asymptomatic atopics compared with either symptomatic atopic or non-atopic individuals [21]. When exposing *in vitro *monocyte-derived dendritic cells with house dust mite Dermatophagoides pteronyssinus 1, functionally active IDO decreased in cells from patients with house dust mite-sensitive asthma compared to non-atopic asthmatics [15]. In asthmatic patients, the baseline IDO activity in sputum has been shown to be significantly lower than control levels, but normal baseline activity in asthmatic patients could be induced by using inhaled corticosteroids [22]. We have previously demonstrated that a high expression of epithelial and leukocyte IDO is associated with chronic rhinosinusitis with nasal polyps, while the expression in chronic rhinosinusitis without nasal polyps remained at the control level [23]. Thus in the upper airways, in contrast to lower airways, it seems that IDO might have a role in the nasal polyp pathomechanisms instead of atopy. Yet, more studies are required to bring evidence on this.

The expression of IDO differs between tissues [24]. Recently, two isoforms of IDO have been studied, IDO1 and IDO2. The expression and activity of both isoform differs in different tissues and in same cell cultures [25]. Both isoforms therefore might differ somehow in their roles [26,27]. Having this in mind, it could be possible that the anti-IDO mAb we used could be specific only for the IDO1 isotope [27]. Also, in some tissues IDO expression does not correlate with its activity. Indeed, elevated expression of IDO can translate into little activity or no activity at all [24]. Even though high levels of IDO expression can be measured, this does not necessarily translate into activity measured by ratio of tryptophan over kynurenine.

Both atopic asthma and atopic rhinitis are complex diseases with different phenotypes and genetic backgrounds. Currently it is thought that patients with certain (epi)genetic elements are prone to have immunological dysfunctions of respiratory barriers putatively by stimulation by certain environmental interactions [28]. Thus, our findings suggest that adult patients with only birch pollen AR but not asthma symptoms do not have local IDO. In the future, it would be of interest to observe whether immunological factors differ in patients having only the AR phenotype compared to those with the AR and asthma phenotype [29].

## Conclusions

This study showed that in the nasal cavity, epithelial and leukocyte IDO expression is low and is not associated to allergic rhinitis caused by birch pollen. There is a controversy in the role of IDO in the pathogenesis of atopy, between upper and lower airways. More human studies are warranted to study the role of IDO in more detail in allergic rhinitis and other atopies.

## Abbreviations

AR: allergic rhinitis; IDO: Indoleamine 2,3-dioxygenase; Ig: immunoglobulin; Th1: T-helper cell 1; Th2: T-helper cell 2.

## Competing interests

The authors declare that they have no competing interests.

## Authors' contributions

ST-S designed the study and collected the specimens with ML; TH performed staining. AL, ST-S, TP, and JK evaluated the specimens. AL and ST-S were involved in the data interpretation, literature search and writing. All authors commented critically on the writing. All authors have read and approved the final manuscript.
